# Anxiety—depression: a pivotal mental factor for accelerating disease progression and reducing curative effect in vitiligo patients

**DOI:** 10.3389/fpsyg.2024.1454947

**Published:** 2024-09-20

**Authors:** Cheng Cao, Fuquan Lin, Rong Jin, Jindi Lei, Yujie Zheng, Anqi Sheng, Wen Xu, Aie Xu, Miaoni Zhou

**Affiliations:** ^1^Department of Dermatology, Hangzhou Third Hospital Affiliated to Zhejiang Chinese Medical University, Hangzhou, China; ^2^School of Basic Medical Sciences, Zhejiang Chinese Medical University, Hangzhou, China

**Keywords:** vitiligo, observational study, anxiety, depression, Mendelian randomization

## Abstract

**Objective:**

To clarify the causal relationship between anxiety-depression and the progression and outcomes of vitiligo, providing a basis for enhancing psychological interventions in the treatment of vitiligo.

**Methods:**

A two-sample Mendelian randomization analysis was used to validate the causal relationship between anxiety, depression, and vitiligo. The Hospital Anxiety and Depression Scale (HADS) assessed the psychiatric condition of vitiligo patients. Fisher’s chi-square tests and rank sum tests analyzed the differences in clinical characteristics among vitiligo patients with different HADS scores. Regression analysis assessed the correlation between anxiety-depression and disease progression and treatment outcomes.

**Results:**

Mendelian randomization analysis showed that depression significantly increases the risk of vitiligo (OR = 4.46, 95% CI: 1.16–17.18, *p* = 0.03). According to the HADS scores, clinical characteristics differed significantly among the groups (*p* < 0.05). Univariate regression analysis demonstrated that both HADS (*B* = –1.168, *p* < 0.001, 95% CI: −1.532 to −0.804) and VIDA (*B* = –2.157, *p* < 0.001, 95% CI: −3.464 to −0.887) were significantly negative associated factors for disease outcomes. However, HADS (*B* = –1.006, *p* < 0.001, 95% CI: −1.475 to −0.657) emerged as the only factor influencing therapeutic efficacy in multivariate regression analysis. A restricted cubic spline graph illustrates a U-shaped relationship between HADS and clinical efficacy, with the most significant impact on therapeutic efficacy occurring when HADS equals 15.

**Conclusion:**

Anxiety—depression exhibit a bidirectional positive causal relationship with vitiligo. Moderate to severe anxiety-depression significantly influences the clinical efficacy of vitiligo treatment.

## 1 Introduction

Vitiligo is a chronic autoimmune skin disease with a global prevalence of approximately 0.5–2%. Its pathogenesis involves multiple factors leading to localized overactive immune responses, which cause specific damage to melanocytes and result in their functional loss. This process leads to localized or generalized depigmentation and the appearance of white patches (Marchioro et al., 2022; Chang et al., 2021). The disfiguring nature of vitiligo significantly impacts the physical and mental well-being of affected individuals. Among these impacts, depression is the most common comorbid condition, correlating linearly with the duration of the disease and significantly reducing the quality of life for patients with vitiligo (Ezzedine et al., 2021; Ning et al., 2022; Simons et al., 2020).

Anxiety—depression are the main comorbid psychosomatic disorders associated with vitiligo. However, the causal relationship between anxiety, depression, and the onset of vitiligo remains unclear. Additionally, their impact on the clinical outcomes of vitiligo treatment has not been explored sufficiently. Studies have indicated that mental health disorders can induce and worsen skin conditions, such as psoriasis and atopic dermatitis, via the brain-skin axis (Marek-Jozefowicz et al., 2022; Chen and Lyga, 2014). Vitiligo patients exhibit a higher incidence of stressful life events prior to the onset of their condition (Picardi et al., 2003). The presence of depression-related polymorphisms in the BDNF and CRH genes and the elevated expression of inflammatory factors in the blood of individuals with depression provide evidence that anxiety—depression could not only result from the disfiguring appearance of vitiligo but could also play a significant role in the induction or exacerbation of the condition, thus affecting treatment outcomes (Kussainova et al., 2022; Patel et al., 2017).

However, there is a lack of large-scale objective evidence to clearly establish the causal relationship between anxiety, depression, and the onset of vitiligo, as well as the influence of anxiety—depression on the clinical characteristics and treatment outcomes of vitiligo.

In this study, we used two-sample Mendelian randomization (MR) analysis, utilizing genetic variations as instrumental variables. This approach leverages the natural random allocation of genetic variations to reduce potential confounding factors (Zuber et al., 2022). We then used the Hospital Anxiety and Depression Scale (HADS) to create a model depicting the influence of anxiety—depression on vitiligo progression and therapeutic outcomes. In this model, HADS scores and the Vitiligo Disease Activity index (VIDA) mutually reinforce each other, with the severity of anxiety—depression negatively impacting the therapeutic efficacy of vitiligo. This negative effect is consistent regardless of gender, subtype, or affected body regions. The progression of vitiligo and clinical characteristics, including sex, disease duration, age, and affected surface area, collectively affect treatment outcomes by influencing the level of anxiety and depression in patients. We identified that individuals with HADS scores in the range of 12–15 experience the most significant influence of anxiety—depression on treatment efficacy, potentially making them the most responsive group to enhanced psychological interventions. This study demonstrates the significant role of anxiety—depression in controlling the progression of vitiligo and achieving repigmentation, providing a solid foundation for improving psychological interventions in the treatment of vitiligo.

## 2 Materials and methods

### 2.1 Mendelian randomization analysis

We obtained data for depression and vitiligo from the Integrated Epidemiology Unit Genome-Wide Association Study (IEU OpenGWAS project) database^1^. The dataset included 460,702 cases of anxiety (Dataset: ukb-b-18336^2^), 170,756 cases of depression (Dataset: ieu-b-102^3^), and 131 cases of vitiligo (Dataset: finn-b-L12_VITILIGO^4^). Ethical approval and informed consent were obtained in the respective original studies.

In this study, we utilized data from the IEU Open GWAS to minimize the impact of weak instrumental variables. To ensure the independence of instrumental variables and eliminate the influence of linkage disequilibrium (LD) on the results, we set a genetic distance threshold of 10,000 kb, an LD threshold of *r*^2^ = 0.01, and a *p*-value threshold of 5 × 10^−8^.

The validation of the relationship between depression and vitiligo primarily employed four regression models: MR-Egger regression, Inverse Variance Weighting (IVW) with random effects, the weighted median method, and the weighted mode method. We also used MR-Egger intercept tests to assess horizontal pleiotropy, Cochran’s *Q* test to evaluate SNP heterogeneity, and the leave-one-out sensitivity test for sensitivity analysis. All mentioned methods were implemented using the TwoSampleMR package in R 4.0.4 software, with a significance level of *α* = 0.05.

### 2.2 Observational study

#### 2.2.1 Study design

A regional, large-scale observational study investigated the association of psychological stress with vitiligo disease progression and clinical outcomes. The protocol was approved by the institutional review board at Hangzhou Third People’s Hospital, and the study was conducted in accordance with Good Clinical Practice guidelines and the ethical principles of the Declaration of Helsinki. Informed consent was obtained from all patients prior to the trial initiation (Ethics approval number and record number: 2023KA037, MR-33-23-034466).

#### 2.2.2 Participants

Patients were eligible for the study if they met the following criteria: (1) diagnosed with vitiligo at the dermatology outpatient department of the Third People’s Hospital in Hangzhou between February 2023 and April 2023; (2) able to complete the questionnaire themselves or with the help of a companion; (3) agreed to the use of their data for clinical research and academic reports. The key exclusion criteria were: (1) inability to complete the questionnaire; (2) refusal to use their data for clinical research and academic publication; (3) presence of high blood pressure, heart disease, severe neuropsychiatric disorders, or impaired consciousness; (4) having a malignant tumor or currently receiving treatment for HIV infection.

#### 2.2.3 Sample size

As our study was observational, we aimed to maximize the sample size while meeting the inclusion criteria. A total of 1,000 samples were registered, and 782 were included in the study after excluding those with irregular or incorrect questionnaire responses.

#### 2.2.4 Assessment of anxiety—depression

We used the Hospital Anxiety and Depression Scale (HADS) to assess anxiety—depression levels in these patients. HADS is a reliable method used by hospitals to evaluate anxiety—depression status (Wondie et al., 2020). We downloaded a publicly available HADS questionnaire and conducted a survey among vitiligo patients with the help of different dermatologists. Patients unable to answer the questions accurately and clearly were excluded. For patients younger than 14 years old or older than 60 years old who could not respond to the questionnaire themselves, family members or companions answered on their behalf based on the patient’s status. During the survey, we asked each question three times and recorded the responses from the patients, their families, or companions. According to the HADS grading criteria, scores of 0–7 indicate a normal status, 8–11 indicate mild anxiety—depression, and scores above 12 indicate moderate to severe anxiety—depression. Clinical efficacy was evaluated based on patient descriptions and the vitiligo area scoring index.

#### 2.2.5 Statistical analysis of the data

Statistical analysis was performed using SPSS 25.0 software. Differences in clinical characteristics among vitiligo patients with varying HADS scores, as well as treatment efficacy, were assessed using the Kruskal–Wallis test and the Chi-squared Fisher test for multiple-group comparisons. The correlation and causal relationships between HADS scores, the Vitiligo Disease Activity Index (VIDA), and clinical efficacy were analyzed using univariate and multivariate linear regression analyses. A significance level of *p* < 0.05 was established for statistical significance.

## 3 Results

### 3.1 Mendelian randomization confirms a positive causal relationship between anxiety, depression and the onset of vitiligo

**Figure 1:**
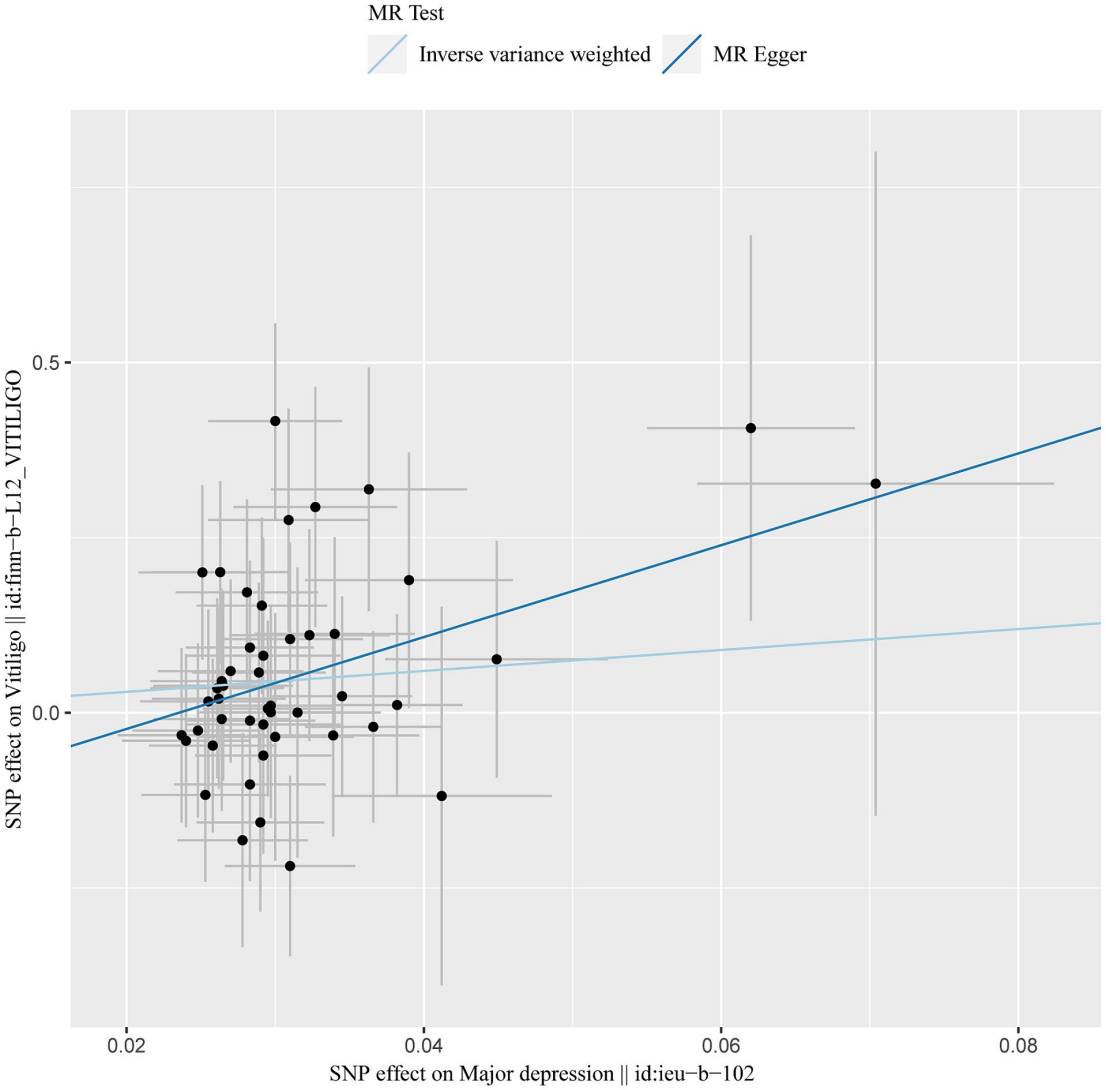
MR results of depression and vitiligo: scatter plot of depression and vitiligo using different MR methods. The slopes of line represent the causal effect of each method, respectively.

**Figure 2:**
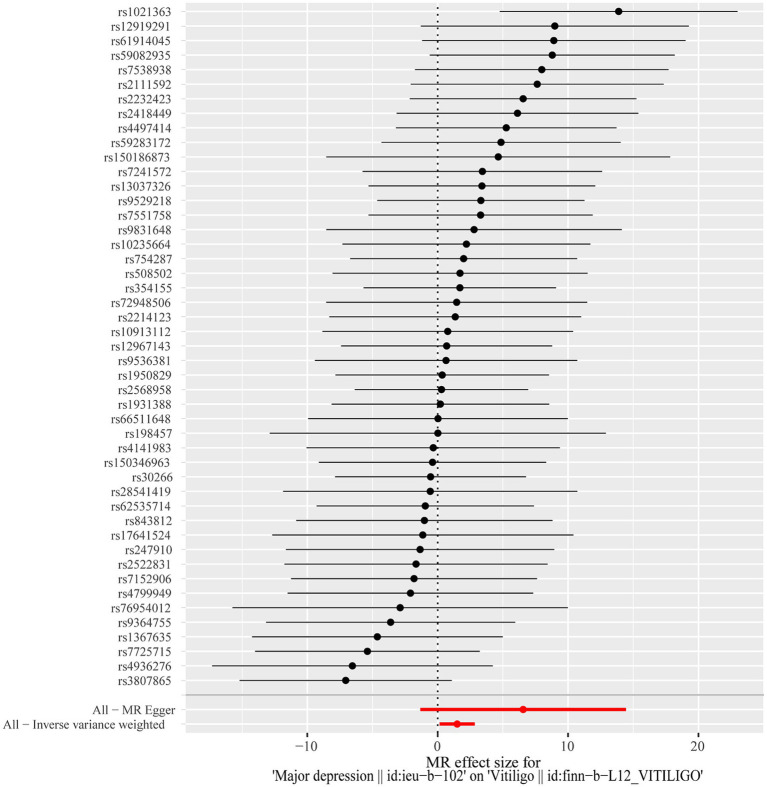
Forest plot of the causal effects of depression associated SNPs on vitiligo. The red and black dot/bar indicate the causal estimate of depression on risk of patients with vitiligo.

**Figure 3:**
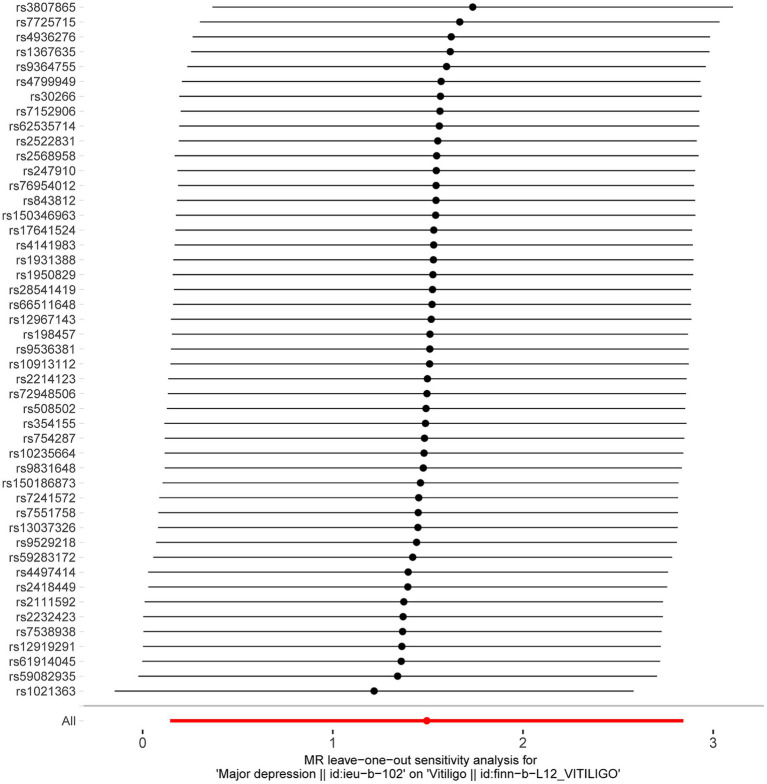
MR results of leave-one-out sensitivity analysis for depression and vitiligo.

After excluding SNPs with a linkage disequilibrium parameter (r^2^ > 0.01) and SNPs not present in the outcome GWAS, 47 SNPs were selected as instrumental variables for the two-sample Mendelian randomization analysis, with all *F* statistics exceeding 10. Initially, no significant association was found between anxiety and the onset of vitiligo (Appendix Table A1). However, the IVW analysis demonstrated a positive causal relationship between depression and vitiligo. The risk of vitiligo in people with depression was 4.46 times higher than in non-depressed individuals, with statistically significant differences (OR = 4.46, 95% CI: 1.16–17.18, *p* = 0.03) (Table 1). To ensure the reliability of the model, a funnel plot was generated, which did not exhibit a typical inverted funnel shape (Figures 1, 2). The Cochran *Q* value indicated no significant heterogeneity between the SNPs for both the MR-Egger method (*Q* = 35.37, *p* = 0.85) and the IVW method (*Q* = 36.99, *p* = 0.83), suggesting minimal susceptibility to bias and underscoring the stability of the model (Table 1). Sensitivity analysis using the leave-one-out method showed minimal fluctuations in the general error lines, with no influential SNP loci identified, further confirming the reliability of the results (Figure 3).

**Table 1:** Mendelian randomization (MR) analysis and sensitivity analysis.

Mendelian randomization (MR) analysis						
Exposure	Outcome	MR. Method	SNP(n)	OR	*P*	95% CI
Depression	Vitiligo	MR Egger	47	703.06	0.11	−1.30 ~ 14.45
Depression	Vitiligo	Weighted median	47	1.96	0.47	−1.13 ~ 2.48
Depression	Vitiligo	IVW	47	1.96	0.03^*^	0.15 ~ 2.84
Depression	Vitiligo	Weighted mode	47	1.36	0.87	−3.38 ~ 4.00

Sensitivity analysis				
Exposure	Outcome	MR. Method	Q	P
Depression	Vitiligo	MR Egger	35.37	0.85
Depression	Vitiligo	IVW	36.99	0.83

SNP, single nucleotide polymorphism; MR, Mendelian randomization; IVW, inverse variance weighted; OR, odds ratio; CI, confidence interval.

### 3.2 Assessment of anxiety—depression status in vitiligo patients

The HADS is a widely used psychological assessment tool in hospital settings. In this study, HADS was used to assess anxiety and depression levels in 1,000 patients with vitiligo. Of these, 782 patients provided valid evaluations, including 338 men (43.2%) and 444 women (56.8%), with ages ranging from 3 to 79 years (clinical details are presented in Table 2). According to the HADS rating criteria, among the 782 vitiligo patients with valid assessments, 198 (25.32%) were classified as having mild anxiety—depression, while 315 (40.28%) were classified as having moderate to severe anxiety—depression. This indicates that more than 65% of patients with vitiligo exhibit varying degrees of comorbid anxiety—depression, highlighting the significant comorbidity of these psychosomatic disorders with vitiligo.

**Table 2:** Descriptive statistics for included participants.

Variables	
Age (years), mean ± SD	29.16 ± 17.14
Disease duration (months), mean ± SD	55.31 ± 67.17
BSA, mean ± SD	0.83 ± 2.24
VIDA, mean ± SD	1.11 ± 1.58
Repigmentation, mean ± SD	31.49 ± 29.21
Gender, *n* (% total)	
Male	444 (56.8%)
Female	338 (43.2%)
Type, *n* (% total)	
Segmental	295 (37.7%)
Non-segmental	487 (62.3%)
Location, *n* (% total)	
Exposed	456 (58.3%)
Non-exposed	326 (41.7%)

SD, standard deviation; BSA, body surface area; VIDA, the vitiligo disease activity score. A total VIDA score of > 1point indicates that the disease is in the progressive stage, while a score of > 4 points indicates that the disease is in the rapidly progressive stage.

### 3.3 Anxiety—depression levels impact clinical characteristics, disease progression, and treatment efficacy in vitiligo patients

Specifically, the moderate to severe anxiety—depression group (HADS≥12) exhibited an older average age (*F* = 8.668, *p* < 0.001), a longer average duration of the disease (*F* = 6.760, *p* = 0.001), a larger mean body surface area (BSA) affected by vitiligo (*F* = 4.605, *p* = 0.010), an increase in the average VIDA from 0.6 ± 1.5 to 2.0 ± 1.5 (*F* = 112.084, p < 0.001), and a significant reduction in the repigmentation rate (χ^2^ = 22.204, p < 0.001). This suggests that moderate to severe anxiety—depression significantly influence the clinical characteristics of vitiligo patients, particularly in disease progression and treatment efficacy. However, the type of vitiligo and the affected body regions did not show statistically significant differences between the three groups (Table 3). These results indicate that moderate to severe anxiety—depression can substantially affect the clinical characteristics of vitiligo, promoting disease progression and reducing the effectiveness of treatment.

**Table 3:** Clinical characteristics of vitiligo patients with different scores.

Variables	Total	HADS ≤ 7	8<HADS<11	HADS ≥ 12	Statistic	*P*
Number, *n*	782	269 (34.4%)	198 (25.3%)	315 (40.3%)		
Age(years), mean ± SD	29.16 ± 17.14	25.95 ± 19.10	29.29 ± 16.62	31.81 ± 15.17	*F* = 8.668	<0.001^***^
Disease duration(months), mean ± SD	55.31 ± 67.17	47.47 ± 55.08	49.02 ± 62.92	65.96 ± 77.19	*F* = 6.760	0.001^***^
BSA (mean ± SD)	0.83 ± 2.24	0.51 ± 1.10	0.89 ± 3.68	1.07 ± 1.68	*F* = 4.605	0.010^**^
VIDA Mean ± SD	1.11 ± 1.58	0.59 ± 1.45	0.37 ± 1.13	2.02 ± 1.50	*F* = 112.084	<0.001^***^
Repigmentation (%), mean ± SD	29.16 ± 17.14	25.95 ± 19.10	29.29 ± 16.62	31.81 ± 15.17	*F* = 26.72	<0.001^***^
Gender, *n* (%)					χ^2^ = 5.861	0.053^*^
Female	444 (56.8)	141 (52.4)	108 (54.6)	195 (61.9)		
Male	338 (43.2)	128 (47.6)	90 (45.5)	120 (38.1)		
Type, *n* (%)					χ^2^ = 3.411	0.19
Segmental	295 (37.72%)	114 (42.38%)	83 (41.92%)	98 (31.11%)		
Non-segmental	487 (62.28%)	155 (57.62%)	115 (58.08%)	217 (68.89%)		
Location, *n* (%)					χ^2^ = 3.637	0.16
Exposed	456 (58.31%)	140 (52.04%)	110 (55.56%)	206 (65.40%)		
Non-exposed	326 (41.69%)	129 (47.96%)	88 (44.44%)	109 (34.60%)		

SD, standard deviation; BSA, body surface area; VIDA, the vitiligo disease activity score. A total VIDA score of > 1point indicates that the disease is in the progressive stage, while a score of > 4 points indicates that the disease is in the rapidly progressive stage.

### 3.4 Bidirectional promotion of anxiety, depression, and vitiligo disease progression

The VIDA is a primary assessment criterion for the onset and progression of vitiligo, with VIDA≥ + 1 indicating an active disease state. Our previous findings demonstrated that the average VIDA score in the moderate to severe anxiety—depression group of vitiligo patients reached 2.0 ± 1.5, significantly higher than that of patients with normal psychological status. A univariate regression analysis further indicated a significant positive effect of HADS on VIDA scores (*B* = 0.121, *p* < 0.001, Table 4). This suggests that anxiety—depression play a crucial role in driving the progression of vitiligo. Conversely, a multivariate regression analysis with HADS as a dependent variable revealed that age, duration of the disease, BSA affected by vitiligo, and VIDA were positively correlated with HADS. However, the relationship between VIDA and HADS scores was the most significant (*B* = 1.413, *p* < 0.001, Table 4). This means that various factors influence anxiety—depression levels in vitiligo patients, with disease progression being the most influential factor. Consequently, it is evident that anxiety—depression in vitiligo patients promote disease progression, while disease progression, in turn, exacerbates anxiety—depression. This reciprocal causation leads to a significant reduction in treatment efficacy, particularly in cases of moderate to severe anxiety—depression.

**Table 4:** Linear-regression analysis showing associations between risk factors and HADS, VIDA.

Variables	*B*	*P*	β (95%CI)
HADS^†^	0.12	<0.001^***^	0.426 (0.104 ~ 0.141)
Disease duration^‡^	0.1	0.024^*^	0.075 (0.001 ~ 0.011)
Age^‡^	0.03	0.001^**^	0.107 (0.013 ~ 0.055)
BSA^‡^	0.16	0.049	0.063 (0.001 ~ 0.31)
VIDA^‡^	1.41	<0.001^***^	0.407 (1.194 ~ 1.633)

^†^Single linear regressions showing associations between HADS and VIDA. ^‡^Multivariate linear regressions showing associations between risk factors and HADS. β, standardized beta coefficient; CI, confidence interval.

### 3.5 Anxiety—depression: significant impacting vitiligo clinical efficacy

Using a univariate regression model, we further explored the influence of anxiety—depression on the clinical efficacy of vitiligo treatment. The results revealed a significant negative association between HADS, VIDA, and clinical efficacy. Specifically, for each one-point increase in HADS, clinical efficacy decreased by 1.168% (*B* = –1.168, *p* < 0.001, 95% CI: −1.532 to −0.804). Similarly, for each one-point increase in VIDA, clinical efficacy decreased by 2.157% (*B* = –2.157, *p* < 0.001, 95% CI: −3.464 to −0.887) (Table 5). We conducted a multivariate regression analysis including covariates, which confirmed a significant negative relationship between HADS and clinical efficacy (*B* = –1.006, *p* < 0.001, 95% CI: −1.475 to −0.657). However, the impact of VIDA on clinical efficacy was not statistically significant (*B* = –0.543, *p* = 0.447, 95% CI: −1.944 to 0.859) (Table 5). Based on our earlier analyses, factors such as VIDA, patient age, sex, duration of the disease, and affected body surface area are critical contributors to increasing the HADS score in patients with vitiligo. Therefore, we conclude that the HADS score is a key factor influencing vitiligo clinical efficacy, while the impact of VIDA on clinical efficacy may be realized by exacerbating patient anxiety—depression.

**Table 5:** Linear-regression analysis showing associations between risk factors and repigmentation.

Variables	*B*	*P*	β (95%CI)
HADS^†^	−1.168	<0.001^***^	−2.200 (−1.532 ~ −0.804)
VIDA^†^	−2.157	<0.001^***^	−1.180 (−3.464 ~ −0.887)
Age^‡^	−0.019	0.761	−0.011 (−0.141 ~ 0.103)
Disease duration^‡^	0	0.989	−0.001 (−0.031 ~ 0.031)
BSA^‡^	−0.657	0.153	−0.05 (−1.557 ~ 0.244)
HADS^‡^	−1.066	<0.001^***^	−0.201 (−1.475 ~ −0.657)
VIDA^‡^	−0.543	0.447	−0.029 (−1.944 ~ 0.859)

^†^Single linear regressions showing associations between risk factors and repigmentation. ^‡^Multivariate linear regressions showing associations between risk factors and repigmentation. β, standardized beta coefficient; CI, confidence interval.

**Figure 4:**
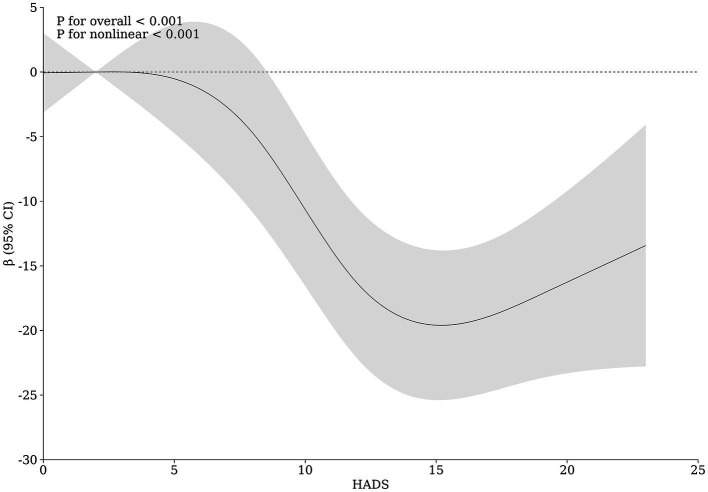
Relationship between HADS and repigmentation in patients with vitiligo.

Further stratified analysis was performed based on sex, affected body regions, and disease type to assess the relationship between anxiety, depression, and clinical efficacy. The results showed that anxiety—depression significantly affected the clinical efficacy of vitiligo patients regardless of gender, body region, and type of disease, without heterogeneity (Table 6). Additionally, the restricted cubic spline graph revealed a positive U-shaped relationship between HADS and clinical efficacy in patients with vitiligo. When HADS scores exceeded 5 points, the impact of HADS on vitiligo clinical efficacy gradually increased, peaking at 15 points (Figure 4). However, once HADS exceeded 15 points, the influence of psychological stress on clinical efficacy gradually diminished.

**Table 6:** Hierarchical analysis of HADS and repigmentation.

	Male	Female
*B*	−0.918	−1.114
*P*	0.006^**^	<0.001^***^
*β* (95%CI)	−0.165 (−1.576 ~ −0.259)	−0.215 (−1.645 ~ −0.583)

	Exposed site	Non-exposed site
*B*	−0.647	−1.584
*P*	0.018^*^	<0.001^***^
*β* (95%CI)	−0.123 (−1.181 ~ −1.113)	−0.292 (−2.236 ~ −0.931)

	Non-segmental	Segmental
*B*	−1.061	−1.088
*P*	<0.001^***^	0.002^**^
*β* (95%CI)	−0.202 (−1.574 ~ −0.547)	0.201 (−1.774 ~ −0.402)

β, standardized beta coefficient; CI, confidence interval.

In summary, anxiety—depression significantly impact the efficacy of vitiligo treatment. Vitiligo patients with HADS scores exceeding 5 points require regular follow-up to monitor their psychological well-being. When HADS scores reach 12–15 points, anxiety—depression can become the primary factors affecting the efficacy of vitiligo treatment, necessitating timely psychological intervention.

## 4 Discussion

Anxiety—depression, among other psychosomatic disorders, are the primary comorbidities of vitiligo and significantly affect the quality of life of those affected. This association is due to the common embryological origin of the skin and brain, both originating in the ectoderm, and the shared regulation of numerous neurotransmitters and hormones in these systems (Marek-Jozefowicz et al., 2022). In particular, Tohid et al. (2019) reported that almost all common mental disorders lead to the onset of certain skin conditions. These skin conditions tend to worsen with stress and are directly related to the course and prognosis of the respective disorders.

Studies have indicated that people with vitiligo are more likely to have experienced stressful life events before the onset of their condition. Factors such as sleep disturbances, stress, lack of support, and unfamiliar environments have been recognized as triggers of vitiligo (Picardi et al., 2003; Papadopoulos et al., 1998; Osinubi et al., 2018). Furthermore, early reports of the effectiveness of antidepressants, such as amitriptyline, in patients simultaneously suffering from vitiligo and depression provide some confirmation of the influence of psychological diseases on vitiligo (Namazi, 2003). In fact, sympathetic disorders have been reported around the lesions in patients with vitiligo, and the onset and course of the skin disease may be significantly affected by stress, mood disorders, or mental disorders (Koga, 1997). Tanwar et al. (2023) reported that catecholamine levels, catecholamine metabolism regulatory genes (COMT and GTPCH1), immune regulatory genes (CTLA4 and PTPN22), and catalase expression were upregulated in the skin and plasma samples of vitiligo patients. These results suggest that there may be sympathetic nervous system disorder-mediated immune inflammation in vitiligo patients around the lesion. Meanwhile, Pang et al. (2014) found that chronic stress could inhibit the formation of skin melanin and the expression level of skin HPA axis factors. When the body is under stress, catecholamine release can be induced through the sympathies-adrenal medulla (SAM) axis, while the skin has peripheral CRH receptors that further promote the release of norepinephrine (Grando et al., 2006). The function of dermal fibroblasts is also affected by epinephrine, including migration and collagen production (Romana-Souza et al., 2011). This process is accompanied by the production and secretion of inflammatory factors, which further induce inflammation and apoptosis of melanocytes, aggravating the development of vitiligo. These findings suggest that anxiety—depression may mediate the release of various hormones and neuropeptides through the HPA axis or the SAM axis, leading to neurogenic inflammation of the skin and inhibiting melanin production. Nevertheless, the extent to which mental factors trigger vitiligo lacks comprehensive quantitative support, and there is limited research on whether they affect the disease’s treatment outcomes. In other words, there is a current lack of substantial research data supporting the importance of psychological interventions in the treatment of vitiligo. Consequently, psychological intervention is often overlooked by dermatologists in the clinical treatment of vitiligo.

This study initially used two-sample Mendelian randomization models to confirm a positive causal relationship between depression and the onset of vitiligo. The findings revealed that people with depression are 4.46 times more likely to develop vitiligo than those without depression, providing the first objective evidence that depression is a risk factor for triggering vitiligo. However, no significant association was found between anxiety and the onset of vitiligo in this study. Subsequently, we assessed the anxiety—depression levels of 782 vitiligo patients using the HADS scale. Through a large-scale investigation and multiple statistical analyses, we explored the impact of anxiety—depression on clinical characteristics and vitiligo treatment outcomes. Our results demonstrated that more than 65% of vitiligo patients experienced varying degrees of anxiety—depression, consistent with previous surveys on the psychological status of vitiligo patients (Simons et al., 2020; Papadopoulos et al., 1998). Among patients with moderate to severe comorbid anxiety—depression (HADS score ≥ 12), significant differences in clinical characteristics were observed, including age, duration of the illness, extent of depigmentation, and progression of the disease, compared to the general population of vitiligo. Furthermore, the treatment results for these patients were significantly inferior to those without significant anxiety—depression. This suggests that anxiety—depression not only increase the risk of vitiligo onset but also play a substantial role in influencing the clinical characteristics and treatment outcomes of vitiligo.

Recently, there has been an increasing application of psychological interventions in the treatment of skin diseases, proving effective in various conditions. Reports have shown that psychological therapy helps eliminate skin symptoms in patients with urticaria, and mental interventions effectively improve treatment outcomes for patients with atopic dermatitis while reducing the use of topical corticosteroids (Simons et al., 2020). These studies emphasize the importance of psychological factors in the treatment of skin diseases and the role of psychological interventions in improving treatment efficacy. This paper provides substantial evidence for the impact of anxiety—depression on the onset, progression, and treatment outcomes of vitiligo. It elucidates the characteristic pattern of how anxiety—depression influence the progression and efficacy of vitiligo treatment. Consequently, it offers a solid theoretical foundation for combined psychological intervention in the clinical treatment of vitiligo. In the future, healthcare professionals may consider incorporating cognitive behavioral therapy, antidepressant medications, talk therapy, support groups, and other psychological interventions in the treatment of vitiligo. This approach can improve the psychological well-being of vitiligo patients and enhance the efficacy of traditional vitiligo treatments.

This study revealed that the HADS has a positive impact on the VIDA. However, HADS is influenced by various factors, including disease progression, age, duration of illness, and extent of depigmentation. Among these factors, the effect of VIDA on HADS was the most significant. Based on these findings, we conclude that HADS and VIDA are positively correlated, indicating a mutual promotion between anxiety—depression and the progression of vitiligo.

Furthermore, HADS scores and the VIDA index were significantly negatively correlated with clinical efficacy. Interestingly, when HADS and VIDA were jointly considered independent variables and age, duration of illness, and extent of depigmentation were included in a multivariate regression analysis, HADS emerged as the only relevant factor affecting treatment outcomes. The influence of HADS on treatment outcomes remained unaffected by gender, type, or site of onset. Consequently, we postulate that the efficacy of vitiligo treatment is significantly influenced by the extent of anxiety—depression. The clinical characteristics and progression of vitiligo indirectly affect treatment outcomes by exacerbating the patient’s anxiety—depression. This result underscores the pivotal role of anxiety—depression in shaping treatment efficacy.

To further delineate the characteristics of anxiety—depression on the efficacy of vitiligo treatment, we constructed a restricted cubic spline plot. The results revealed a U-shaped relationship between the HADS score for vitiligo patients and their clinical results. The influence on treatment efficacy was most pronounced at HADS scores of 15. Furthermore, vitiligo patients with moderate to severe anxiety—depression (HADS ≥ 12) exhibited the most significant differences in clinical characteristics and treatment results compared to the general population of vitiligo. Consequently, we contend that vitiligo patients with HADS scores in the range of 12–15 should be the target population for proactive psychological interventions. Within this group, active psychological intervention has the potential to significantly enhance the efficacy of vitiligo treatment.

## Data Availability

Publicly available datasets were analyzed in this study. This data can be found at: Dataset: IEU OpenGWAS project at https://gwas.mrcieu.ac.uk/datasets/ieu-b-102/ and https://gwas.mrcieu.ac.uk/datasets/finn-b-L12_VITILIGO/.

## Footnotes

^1^https://gwas.mrcieu.ac.uk/

^2^https://gwas.mrcieu.ac.uk/datasets/ukb-b-18336/

^3^https://gwas.mrcieu.ac.uk/datasets/ieu-b-102/

^4^https://gwas.mrcieu.ac.uk/datasets/finn-b-L12_VITILIGO/

## Appendix

**Table A1:** Mendelian randomization (MR) analysis and sensitivity analysis, anxiety and vitiligo.

Mendelian randomization (MR) analysis						
Exposure	Outcome	MR. Method	SNP(n)	OR	*P*	95% CI
Anxiety	Vitiligo	MR Egger	5	6.27E+76	0.07	47.66 ~ 306.00
Anxiety	Vitiligo	Weighted median	5	16914.90243	0.61	–27.87 ~ 47.34
Anxiety	Vitiligo	IVW	5	2.31316E+11	0.20	–13.67 ~ 66.01
Anxiety	Vitiligo	Weighted mode	5	10.61	0.93	–45.93 ~ 50.65

Sensitivity analysis				
Exposure	Outcome	MR. Method	*Q*	*P*
Anxiety	Vitiligo	MR Egger	2.43	0.49
Anxiety	Vitiligo	IVW	7.92	0.09

SNP, single nucleotide polymorphism; MR, Mendelian randomization; IVW, inverse variance weighted; OR, odds ratio; CI, confidence interval.
